# A Case Report of Probable Sporadic Creutzfeldt-Jakob Disease: How to Approach Early Diagnosis?

**DOI:** 10.7759/cureus.1297

**Published:** 2017-05-30

**Authors:** Bowei Tan, Carlos Morales Mangual, Iftekhar Mahmud, Nosakhare D Tongo, Larisa Mararenko, Arthur Kay

**Affiliations:** 1 Department of Medicine, Brookdale University Hospital and Medical Center; 2 Department of Neurology, Brookdale University Hospital and Medical Center

**Keywords:** sporadic creutzfeldt-jakob disease, akinetic mutism, mri, electroencephalogram, protein 14-3-3

## Abstract

Sporadic Creutzfeldt-Jakob disease (sCJD) is a rare and fatal spongiform encephalopathy characterized by rapidly progressive dementia and myoclonus. The rarity of this disease and varied initial symptoms make the early diagnosis fairly challenging. Here, we present a case initially admitted for confusion and bizarre behaviors. She had acute deterioration of mental status, akinetic mutism, and myoclonus jerks four weeks later. Cerebrospinal fluid (CSF) analysis was positive for protein 14-3-3. Brain magnetic resonance imaging (MRI) showed hyperintensities in the bilateral cortex, basal ganglia, and thalami in diffusion-weighted imaging (DWI). Electroencephalogram (EEG) showed bihemispheric periodic lateralizing epileptiform discharges. The probable diagnosis of sCJD was reached based on the clinical features, characteristic findings in her MRI, the EEG, and a positive 14-3-3 CSF assay. The literature was also reviewed for early diagnosis of sCJD.

## Introduction

Creutzfeldt-Jakob disease (CJD) is a transmissible, progressive, and fatal human prion disease characterized by rapidly progressing dementia and myoclonus. It can be divided into four categories: sporadic (sCJD), familial (fCJD), iatrogenic (iCJD), and variant forms (vCJD). sCJD accounts for 85-95% of total CJD cases and the incidence is about 1 - 1.5 people per million annually; 10% of CJD cases are familial CJD, and iatrogenic and variant forms compose of 2-5% CJD cases [1]. sCJD is always suspected when patients have significant rapid progressive dementia, cerebellar function abnormalities, and pyramidal/extrapyramidal signs. However, aberrant behaviors, such as anxiety, irritability, social withdrawal, subtle changes in memory, judgment difficulties, and other psychiatric symptoms, are frequently reported as early signs but can be easily neglected [2]. Here, we report a case of sCJD that initially presented with cognitive impairment, behavioral changes, and extrapyramidal signs; the patient's dementia progressed rapidly and followed with myoclonic jerks and akinetic mutism at the end stage. 

## Case presentation

A 58-year-old African American female was brought to Brookdale Hospital emergency room (ER) after she was found wandering in the street wearing net stockings, one sock, and a sleeveless dress on a cold winter night. She was confused, disheveled, anxious, and shivering upon admission. Later, she became agitated, combative, kept speaking coarse vulgar words, and spat at the medical staff. She had been living an isolated life and was estranged from family members and friends. She admitted that she had a “memory problem” for a while but denied any history of other medical issues and/or psychiatric diseases. She denied any neurosurgery history. Family history was not contributory.  

General physical examination was unremarkable. Neurological examination showed a significant cognitive impairment and cerebellar signs with limb and gait ataxia. Motor and sensory functions were within normal. She didn't show apraxia, acalculia, aphasia, or other cortical dysfunctions. Dementia workup, including thyroid function, syphilis testing, vitamin B12 level, and human immunodeficiency virus screening, was insignificant. Computed tomography (CT) scan of the head did not show any hydrocephalus or acute/old infarcts. Laboratory testing was consistent with prerenal azotemia, which normalized in 24 hours with intravenous hydration. She had intermittent episodes of agitation, confusion, and verbal abusive behaviors at night time during hospitalization. The patient was diagnosed with early onset dementia and discharged to a nursing home.

**Figure 1:**
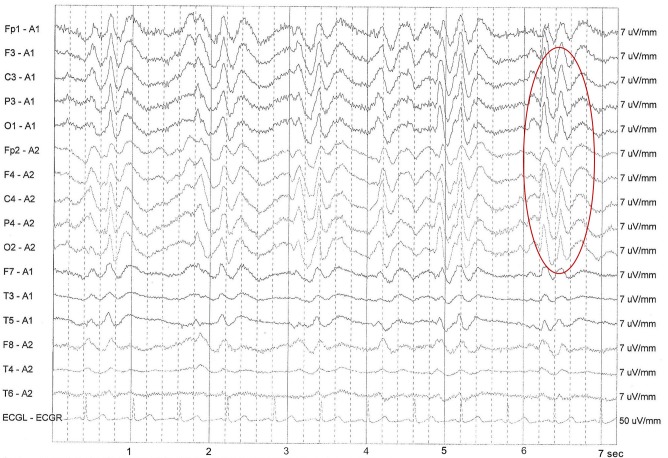
Periodic synchronous biphasic sharp wave complex (PSWC) in electroencephalography (red circle).

**Figure 2:**
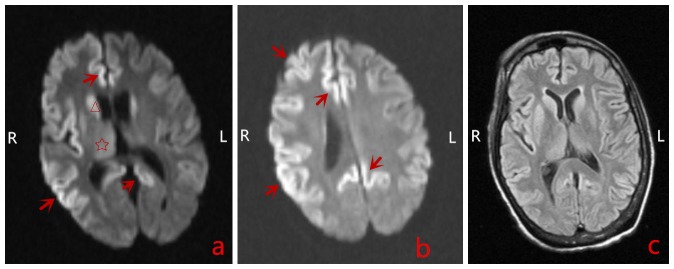
Brain magnetic resonance imaging (MRI) study a) Diffusion-weighted imaging (DWI) showed increased abnormal signal intensities with restricted diffusion in the cerebral cortex bilaterally (arrows, Right > Left), basal ganglia (triangle), and thalami (asterisk); b) DWI showed abnormal hyperintensities with restricted diffusion in the bilateral cerebral cortex (arrows, Right > Left); c) Fluid-attenuated inversion recovery imaging: relatively normal brain MRI imaging.

Four weeks later, the patient was readmitted due to acute deterioration of mental status, failure to thrive, and mutism. She became completely mute and bed-bound. Neurological examinations were significant for spastic contractions of arms and legs, diffuse myoclonic twitching with marked startle response, and no deep tendon reflexes elicited. sCJD was suspected with the typical akinetic mutism, rapid progressive cognitive impairment, and myoclonus. CSF analysis was negative for infection but positive for protein 14-3-3. EEG revealed typical findings of bihemispheric periodic lateralizing epileptiform discharges (Figure 1). Brain MRI showed extensive high intensities with restricted diffusion in the bilateral cortex, basal ganglia, and thalami in diffusion-weighted imaging (DWI) (Figure 2a, b) and a normal appearance in fluid-attenuated inversion recovery (FLAIR) imaging (Figure 2c). The probable diagnosis of sCJD was made based on the clinical features, characteristic findings of the MRI, the EEG, and a positive 14-3-3 CSF assay. The patient was discharged to hospice in the nursing home and expired one month later; an autopsy was not performed.

## Discussion

It is of great prognostic value to diagnose sCJD at the first clinical encounter so that it does not fall into the category of treatable dementia. However, to establish the diagnosis of sCJD in the early stages is fairly challenging due to the extremely low incidence and highly varied initial symptoms. Other rapidly progressive dementias can also resemble sCJD because of the overlap of motor, behavioral, psychiatric, and cognitive manifestations, which make the diagnosis of sCJD in the early stages even more difficult. The concomitant psychiatric symptoms and/or extrapyramidal signs may mask the early onset of dementia. Twenty percent of patients may first manifest with “behavioral symptoms”, such as agitation, irritability, and depression in the early stages [1]. In a study of 248 sCJD cases in Germany, 64% of patients had agitation, 45% had hallucinations, 50% had anxieties, and 37% had depression at the onset of the disease [3]. The myoclonus, especially provoked by startle, may be absent at the first presentation but usually appears in the advanced stages of sCJD [4]. Akinetic mutism is usually manifested at the end stage of sCJD, which is associated with the presence of psychiatric symptoms or cerebellar disturbances as initial symptoms [5].

Here, we present a probable sCJD case with typical symptoms and clinical course, especially at the end stage; however, the correct diagnosis wasn't reached during the early course of the disease. Her cognitive impairment led to the diagnosis of early onset dementia. The initial presentation of acute agitation, confusion, and irritabilities was attributed to acute delirium or neuropsychiatric symptoms of dementia. However, her bizarre behaviors, social withdrawal, and concomitant cerebellar signs were not given enough emphasis, which caused a delay of diagnosis in this case. sCJD should always be included in the differential diagnosis when the diagnosis of dementia is reached on top of significant behavioral disturbance, neurological findings, or psychiatric symptoms. 

The definitive neuropathological diagnosis of sCJD depends on biopsy/autopsy, which is characterized by the loss of neurons, gliosis, spongiform degeneration, or the presence of protease-resistant prion protein (PrPSc) on histopathology of brain tissue. However, this may not be feasible in clinical practice. Therefore, the Centers for Disease Control (CDC) proposed a probable diagnosis of sCJD based on these four criteria: (1) progressive dementia; (2) at least two out of the four following clinical features: a) myoclonus; b) visual or cerebellar disturbance; c) pyramidal/extrapyramidal dysfunction; and/or d) akinetic mutism; (3) a typical electroencephalogram and/or a positive 14-3-3 CSF assay and/or magnetic resonance imaging (MRI) high signal abnormalities on diffusion-weighted imaging (DWI) or fluid-attenuated inversion recovery (FLAIR); and (4) routine investigations for dementia are unremarkable for other alternative diagnoses. In our case, even though neither brain biopsy nor autopsy was done for definitive diagnosis, her clinical features, brain MRI, EEG, and a positive 14-3-3 CSF assay fitted all CDC criteria for the probable diagnosis of sCJD.

DWI and FLAIR imaging of a brain MRI can be utilized for the early diagnosis of sCJD with fairly high sensitivity (91%) and specificity (95%) [6]. The typical and specific diagnostic pattern is gray matter hyperintensities in the cortex, striatum, medial, and/or posterior thalamus. Hyperintensities in the DWI imaging greater than FLAIR imaging and associated restricted diffusion are more common in sCJD than in any other rapidly progressive dementia diseases which may facilitate differentiating sCJD from other prion diseases [7]. EEG can also be used as an adjunctive non-invasive method to diagnose sCJD. The periodic sharp wave complexes (PSWCs) in the EEG are very typical for sCJD patients. The sensitivity of the typical EEG findings is generally low but represents a high specificity if, in fact, it is positive [8]. CSF 14-3-3 protein analysis has a relatively high sensitivity but moderate specificity for the diagnosis of sCJD. In a systematic review of 1,849 patients with suspected sCJD, the assays of CSF 14-3-3 protein possessed a sensitivity of 92% and specificity of 86% in diagnosing sCJD [9].

Real-time quaking-induced conversion (RT-QuIC) was recently described as in vitro amplification technology for detection of PrPSc in CSF. This technique uses recombinant PrP as a substrate added into patient’s CSF samples; the native PrP can convert to PrPSc via intermittent automated shaking, then generate a larger amount of PrPSc that subsequently can be detected by traditional methods. Orrú, et al. used RT-QuIC with olfactory mucosa brushings and showed a 97% sensitivity and 100% specificity for diagnosing sCJD [10].

## Conclusions

sCJD disease should be suspected early in the setting of new-onset dementia with concomitant behavioral disturbances, cerebellar signs, or psychiatric symptoms. Early diagnosis can be established with the clinical features, MRI, EEG, and CSF 14-3-3-protein analysis. RT-QUIC can also be utilized for early definitive diagnosis, if feasible. Also, there is a possibility of iatrogenic transmission when the medical instruments come into contact with the olfactory mucosa of humans with sCJD, which requires further study in the future.
